# Clinical effect of limited posterior decompression and 13-mm titanium mesh implantation on severe thoracolumbar burst fractures: A case series

**DOI:** 10.3389/fsurg.2023.1132569

**Published:** 2023-03-16

**Authors:** Liu Jingcheng, Lu Lei

**Affiliations:** Spinal Surgery, China Medical University Shaoxing Hospital, Shaoxing, China

**Keywords:** thoracolumbar fracture, titanium mesh implantation, spinal canal decompression, case series, thoracolumbar burst fractures

## Abstract

**Background:**

Posterior incision with 270° spinal canal decompression and reconstruction surgery is a treatment option for thoracolumbar burst fractures (TLBF), but the large diameter titanium mesh placement is difficult. This study evaluated the characteristics and clinical effects of limited posterior decompression and 13-mm titanium mesh implantation to treat TLBF.

**Hypothesis:**

13-mm titanium meshes could be used to fix thoracolumbar burst fractures.

**Patients and methods:**

This case series included patients who underwent limited posterior decompression and 13-mm titanium mesh implantation at China Medical University Shaoxing Hospital (01/2015–12/2019). The Cobb angle, injury vertebral anterior edge height loss percentage, and spinal canal occupancy rate were analyzed. The degree of spinal cord injury was evaluated according to the ASIA grade.

**Results:**

Fifteen patients were included (eight males and seven females). The patients were 32.2 ± 4.6 years of age. The American Association of Spinal Injury improved after surgery (A/B/C/D/E: from 2/6/5/2/0 to 0/0/2/8/5, *P* < 0.001). The Cobb angle decreased after surgery (from 20.1 ± 4.8° to 7.1 ± 1.4°, *P* < 0.001) but increased to 8.2 ± 0.9° at 1 year (*P* = 0.003). The percentage of loss of the anterior edge height of the injured vertebrae decreased after surgery (from 40.9% ± 6.1% to 7.5% ± 1.8%, *P* < 0.001) and decreased at 1 year (7.0% ± 1.5%, *P* = 0.044). The spinal canal occupancy rate decreased after surgery (from 64.8% ± 7.8% to 20.1% ± 4.2%, *P* < 0.001) but did not decrease further at 1 year (19.4% ± 3.4%, *P* = 0.166).

**Discussion:**

Spinal canal limited posterior decompression, and 13-mm titanium mesh implantation in the treatment of TLBF can achieve one-stage spinal canal decompression and three-column reconstruction. The curative effect was satisfying.

**Level of evidence:**

Level IV; case series.

## Introduction

Thoracolumbar burst fracture usually results from high-energy (younger patients) or low-energy (older patients) vertebral injury (1). The burst bone fragments can squeeze into the spinal canal, leading to spinal cord compression and impaired nerve function (1, 2).

The proper management of such fractures is crucial since they can easily cause chronic pain (3–6). The treatment goals are to prevent neurological damage, establish stability and fusion of the injured spine, preserve sagittal balance, and initiate early rehabilitation and return to normal activities (1, 2, 7), but how to achieve these goals remains controversial (3–6). The surgical methods for treating thoracolumbar burst fractures include the anterior spinal approach, combined approach, and posterior approach (1, 2, 7). Although the anterior approach can treat fractures of the anterior and middle column of the vertebral body, it cannot treat all injuries to the posterior longitudinal ligament complex and column (1, 2, 7). The anteroposterior combined approach is used to reconstruct the injured vertebra and repair the posterior longitudinal ligament complex. Still, the operation is difficult and time-consuming (1, 2, 7). Therefore, the posterior short-segment pedicle internal fixation is a commonly used treatment method (8), but the posterior approach alone cannot effectively treat anterior and middle column fractures (1, 2, 7).

Many authors advocated a new surgical method for thoracolumbar burst fracture, namely the three-column reconstruction of the vertebral body with the implantation of titanium mesh for one-stage spinal canal decompression (9, 10). In 2008, Huazi et al. (11) described an innovative treatment method for thoracolumbar burst fractures through a posterior incision with 270° spinal canal decompression and reconstruction surgery.

Nevertheless, most of the surgical methods reported in China use titanium meshes with a diameter of 18 or 20 mm, but large-diameter titanium mesh needs to remove more injured vertebrae, with large blood loss during operation, long operation time, and difficulty to place. Small-diameter titanium mesh may have more advantages. From January 2015 to December 2019, many patients with thoracolumbar burst fractures were treated successfully by limited posterior decompression and 13-mm titanium mesh implantation at China Medical University Shaoxing Hospital. To the best of the authors’ knowledge, no previous study reported using 13-mm titanium meshes for thoracolumbar burst fractures. Therefore, this retrospective study aimed to evaluate the characteristics and clinical effect of limited posterior decompression and 13-mm titanium mesh implantation to treat thoracolumbar burst fractures.

## Methods

### Study design and patients

This case series included consecutive patients who underwent limited posterior decompression and 13-mm titanium mesh implantation to treat thoracolumbar burst fractures at the Department of Spinal Surgery of China Medical University Shaoxing Hospital from January 2015 to December 2019. This study was approved by the Ethics Committee of China Medical University Shaoxing Hospital. The requirement for individual informed consent was waived by the committee.

The inclusion criteria were (1) thoracolumbar burst fractures with single-segment vertebral body injury [according to the Three-Column Theory of Denis (6)], (2) Thoracolumbar Injury Classification and Severity Score (TLICS) (12) ≥7 points, (3) Load Sharing Classification (LSC) score (13) ≥7 points, (4) a 13-mm titanium mesh was implanted through the posterior spinal approach, and (5) follow-up time ≥1 year with complete data. The exclusion criteria were (1) multi-segment spinal injury, (2) spinal deformity, (3) autoimmune diseases or any underlying diseases affecting neurological function, (4) very severe osteoporosis, or (5) concomitant cranial nerves or severe thoracic and abdominal joint organ injury.

### Data collection

The routine procedures are presented in the Supplementary Material. The patients’ general characteristics were collected. The patients were re-examined at 1, 3, 6, and 12 months after surgery. Three senior clinicians evaluated the spinal canal occupancy area of the injured vertebra, the percentage of loss of the anterior edge height of the injured vertebra, the Cobb angle, and the American Association of Spinal Injury (ASIA) grade.

### Statistical analysis

SPSS 22.0 (IBM, Armonk, NY, United States) was used for data analysis. Categorical data were presented as *n* (%) and analyzed using Fisher's exact test. Quantitative data were presented as means ± standard deviation and analyzed using repeated-measures analysis of variance with the Bonferroni *post hoc* method. The ASIA grades were analyzed as ordinal data. The Stata/SE 15.1 data analysis package (StataCorp LP, College Station, TX, United States) was used to perform two independent sample *t*-tests on the mean ± standard deviation data of the results of two independent studies, analyzing whether the results of the two independent studies were statistically significant. *P*-value <0.05 indicated statistically significant differences.

## Results

### Characteristics of the patients

Fifteen patients were included. All patients had thoracolumbar burst fractures due to high-energy trauma. At admission, the Cobb angle of the injured vertebra was 20.1 ± 4.7°, the percentage of loss of the anterior edge of the vertebra height was 40.9% ± 5.9%, and the spinal canal occupancy area was 64.8% ± 7.5% (Table 1).

**Table 1:** Characteristics of 15 patients before, after surgery, and at 1-year after surgery.

General information	Patient (*n* = 15)
Sex	
Male (*n*)	8
Female (*n*)	7
Age (year, mean ± SD)	32.2 ± 4.6
Types of high-energy injuries	
Car accidents (*n*)	8
High-altitude falling accidents (*n*)	3
Falling objects injury (*n*)	4
TLBF location	
T12 (*n*)	2
L1 (*n*)	6
L2 (*n*)	6
L3 (*n*)	1
TLICS score (mean ± SD)	7.53 ± 0.71
LSC score (mean ± SD)	7.73 ± 0.77
ASIA grade	
A (*n*)	2
B (*n*)	6
C (*n*)	5
D (*n*)	2
Operation time (min, mean ± SD)	208 ± 21
Intraoperative bleeding (ml, mean ± SD)	900 ± 359
Postoperative negative pressure drainage flow (ml, mean ± SD)	361 ± 122
Inpatient days (day, mean ± SD)	25.7 ± 4.1

TLBF, thoracolumbar burst fracture; TLICS, thoracolumbar injury classification and severity score; LSC, load sharing classification; ASIA, American Association of Spinal Injury.

All patients were followed up after surgery, and their postoperative recovery was good. The spinal sequence and physiological curvature were restored, and the injured spinal canal was fully decompressed (Table 1).

**Figure 1:**
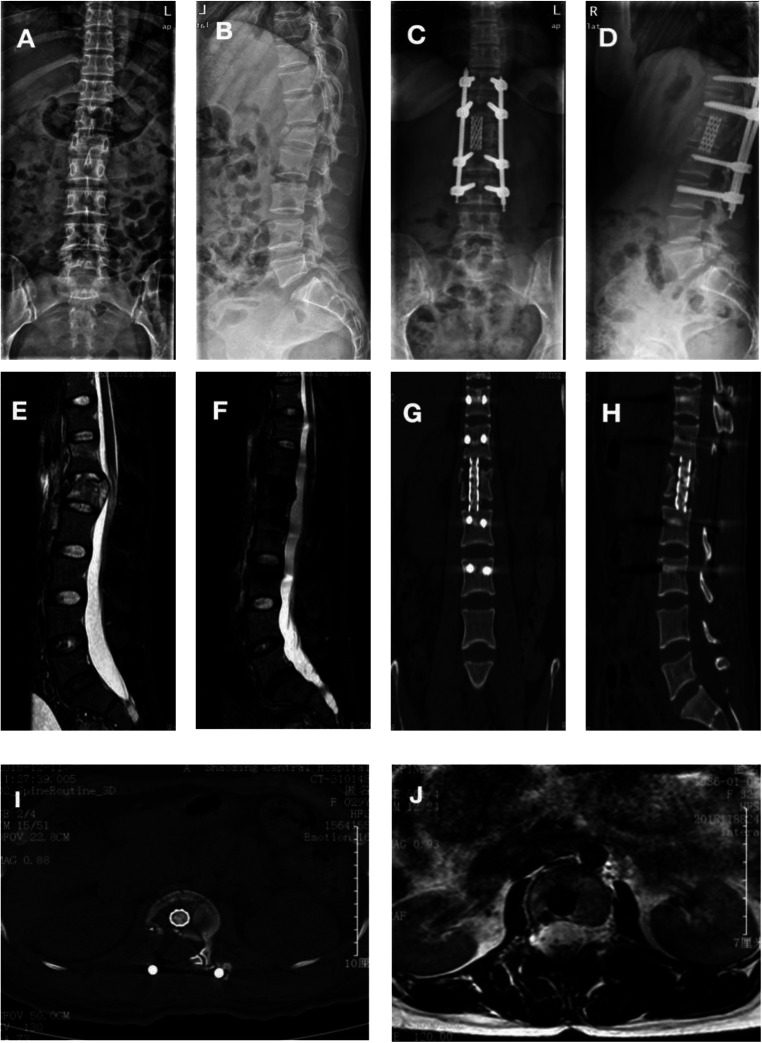
Typical preoperative, postoperative, and follow-up images of severe thoracolumbar burst fractures with posterior midline spinal approach for spinal canal limited decompression and 13-mm titanium mesh implantation (case #1). (**A,B**) True lateral x-rays of the patient's thoracolumbar spine before the operation shows the L1 fracture. (**C,D**) True lateral x-rays of the thoracolumbar vertebrae after the operation showing good fixation and recovery of the height of the anterior edge of the L1 vertebra itself. (**E**) Preoperative magnetic resonance imaging (MRI) shows L1 burst fracture, spinal cord compression, and obvious spinal canal occupancy. (**F–J**) Postoperative MRI indicates no spinal cord compression and no spinal canal occupancy. (**G–I**) Postoperative computed tomography (CT) indicates a good position of the titanium mesh, screws, and titanium rods and good bone graft fusion. (**H**) Thoracolumbar CT after the removal of titanium rods and screws.

**Figure 2:**
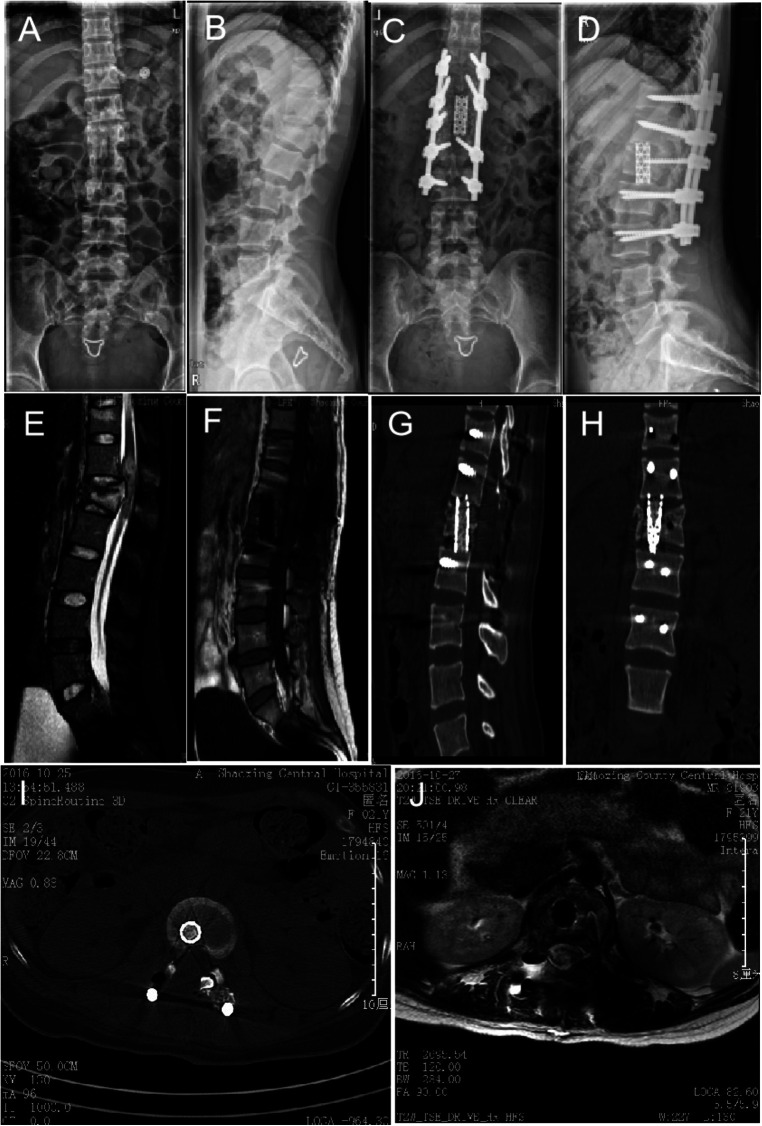
Typical preoperative, postoperative, and follow-up images of severe thoracolumbar burst fractures with posterior midline spinal approach for spinal canal limited decompression and 13-mm titanium mesh implantation (case #2). (**A,B**) True lateral digital x-rays of the patient's thoracolumbar spine before the operation showing the L1 fracture. (**C,D**) True lateral digital x-rays of the thoracolumbar vertebrae after the operation showing good fixation and recovery of the height of the anterior edge of the L1 vertebra. (**E,F**) Preoperative magnetic resonance imaging (MRI) showing L1 burst fracture, spinal cord compression, and obvious spinal canal occupancy. (**G–J**) Postoperative MRI indicates no spinal cord compression and no spinal canal occupancy. (**I**) Postoperative computed tomography (CT) indicates a good position of the titanium mesh, screws, and titanium rods.

The radiological data 1 year after surgery showed fusion of the bone graft in the 13-mm titanium mesh in all patients. There were no space-occupying lesions in the spinal canal of the injured vertebrae and no signs of loosening and falling off of screws and connecting rods during follow-up. The imaging results of typical cases are shown in Figures 1, 2.

### ASIA grade of patients before surgery and 1 year after surgery

The 13 patients performed better than they were at admission (*P* < 0.001) (Table 2). The two grade A patients improved to grade D. In grade B patients, one improved to grade C, three to grade D, and two to grade E. Of the grade C patients, one remained grade C, two improved to grade D, and two improved to grade E. In the two grade D patients, one remained unchanged, and the other improved to grade E (Table 2). One patient with a thoracolumbar burst fracture underwent another surgery 1 year later to remove four pedicle screws and thus freed the range of motion of two vertebral body segments. The postoperative recovery was good. One patient with a thoracolumbar burst fracture was evaluated, and all internal fixation devices were removed 1 year after surgery with good postoperative recovery.

**Table 2:** Comparison of patients’ ASIA grade between pre-operation and 1-year after surgery.

ASIA grade (*n* = 15)				
Index	Pre-operation	One-year after surgery	Statistical value	*P*
ASIA grade (A/B/C/D/E)	2/6/5/2/0	0/0/2/8/5	*X*^2^ = 17.269	0.001

ASIA, American Association of Spinal Injury.

### Comparison of imaging outcomes

The Cobb angle decreased after surgery (from 20.1 ± 4.8° to 7.1 ± 1.4°, *P* < 0.001) but increased a little to 8.2 ± 0.9° at 1 year (*P* = 0.003). The percentage of loss of the anterior edge height of the injured vertebrae decreased after surgery (from 40.9% ± 6.1% to 7.5% ± 1.8%, *P* < 0.001) and decreased a little more at 1 year (7.0% ± 1.5%, *P* = 0.044). The spinal canal occupancy rate decreased after surgery (from 64.8% ± 7.8% to 20.1% ± 4.2%, *P* < 0.001) and did not decrease further at 1 year (19.4% ± 3.4%, *P* = 0.166) (Table 3).

**Table 3:** Comparison of Cobb angle, loss percentage of anterior vertebral height, and spinal canal occupancy rate of 15 patients before operation, after the operation, and at 1-year after surgery.

Variable	Before operation	After operation	1-year after surgery	*P*_Postoperation_ vs. *P*_Preoperation_	*P*_1-year after surgery_ vs. *P*_Postoperation_	*P*_1-year after surgery_ vs. *P*_Postoperation_
Cobb angle (degree, mean ± SD)	20.1 ± 4.8	7.1 ± 1.4	8.2 ± 0.9	<0.001	<0.001	0.003
Loss percentage of anterior vertebral height (%, mean ± SD)	40.9 ± 6.1	7.5 ± 1.8	7.0 ± 1.5	<0.001	<0.001	0.044
Spinal canal occupancy rate (%, mean ± SD)	64.8 ± 7.8	20.1 ± 4.2	19.4 ± 3.4	<0.001	<0.001	0.166

## Discussion

The goals of surgical treatment for patients with a thoracolumbar fracture are to rebuild the biological stability of the injured vertebral body and decompress the spinal canal and avoid neurological dysfunction fully, and eventually improve the patient's quality of life (1, 2, 7). In 1968, thoracolumbar posterior three-column reconstruction was first described to treat a giant cell tumor of the lumbar spine. In 2003, implantation of titanium mesh and posterior spinal canal decompression was performed to treat thoracolumbar burst fractures and achieved satisfactory clinical effects (1, 2, 7). In China, one-stage three-column reconstruction and spinal canal decompression for patients with severe thoracolumbar burst fractures accompanied by spinal cord damage was first carried out by Huazi et al. in 2005 (11).

Burst fractures are caused by an axial plane compression force of the vertebral body (1). The nucleus pulposus of the intervertebral disc protrudes into the spinal canal from the superior endplates, which accounts for 21%–58% of thoracolumbar fractures (14). The objective of one-stage spinal canal surgical treatment is to decompress the dural sac to avoid aggravating the compression of the spinal cord. Based on the three-column theory of Denis (6), the corresponding anterior, middle, and posterior spine columns are reconstructed to ensure the stability of the spine.

For severe thoracolumbar burst fractures, the anterior or posterior approach through the injured vertebra alone cannot completely decompress the injured spinal canal while achieving a three-column reconstruction of the injured vertebral body. The classic surgical methods include posterior spinal approach decompression and pedicle screw fixation (15). It is undeniable that such a surgical method can achieve the reconstruction of the posterior column; however, when the anterior, middle, and posterior columns are all injured, the cancellous bone of the anterior and middle columns cannot be repositioned in one stage, that is, the “eggshell vertebral body”. With the loss of height between the upper and lower endplates of the anterior edge of the injured vertebral after surgery, the physiological curvature of the spine is destroyed, and the internal fixation device of the injured vertebral body fails. Compared with the anterior or posterior approach alone, the combined anteroposterior approach results in increased operation time, intraoperative bleeding, and trauma and thus lead to complex perioperative management (15). Although the anterior approach alone can achieve the operation under direct vision, the spinal canal cannot be decompressed completely, and the posterior column of the injured vertebra cannot be reconstructed. Furthermore, it is difficult to reposition the intervertebral joints. Besides, there are many organs in the thorax and abdomen, leading to serious consequences of postoperative infections.

In order to solve the problems of traditional surgical treatment of thoracolumbar burst fractures and maximize the risk-benefit ratio, Huazi et al. (11). proposed an innovative surgical method: 270° spinal canal decompression and reconstruction surgery through a posterior incision. The potential damage of the operation itself is reduced, and the three columns of the vertebral body are effectively reconstructed. The indications for this operation are severe thoracolumbar burst fractures, three-column integrity destruction, LSC ≥7 points, and good general conditions. In this study, all 15 patients met these surgical indications. The 270° spinal canal decompression and 18-mm large-diameter titanium mesh surgery were used for implementation. The advantages of this surgical method are that the spinal canal of the injured spinal canal was fully relieved from the one-stage operation, and the anterior and middle column was reconstructed with titanium mesh implantation. Secondly, the titanium mesh implantation ensures the stability of the anterior and middle columns of the vertebral body, while the pedicle screw fixation repairs the posterior column. Moreover, titanium mesh implantation distributes the stress of the pedicle screw during the movement of the vertebral body and thus avoids internal fixation failure. Thirdly, after filling and fusion with a fragment of autogenous bone, the intervertebral height is not easily lost and thus reduces the incidence of secondary kyphosis. Fourthly, the patients have a good long-term prognosis. At the same time, the disadvantages of the surgical method are that the 270° lateral anterior curettage of the injured vertebral cancellous bone might lead to massive bleeding. Secondly, it is difficult to measure the intervertebral height during the operation accurately. The larger the diameter of the titanium mesh, the more difficult the implantation is. Meanwhile, there is a risk of nerve root traction, and accordingly, the operation time is prolonged (16). Thirdly, since the operation is complicated, the doctors need to have rich clinical experience and skills. Fourthly, it is difficult to clean the bone fragment completely because of the potential blind spot. Because of the disadvantages described above, such a surgical method has not been widely promoted in China to treat severe thoracolumbar burst fractures.

The published data by Dun et al. (17) (mesh of 18 or 20 mm) were used for comparison. The analysis showed that the operation time was significantly shortened with the 13-mm mesh (*t* = 10.11; *P* < 0.001) and that the volume of bleeding during the operation was significantly reduced (*t* = 2.32; *P* < 0.05), but without difference for the negative pressure drainage volume after surgery (*t* = −0.18; *P* = 0.86). The three-column reconstruction of 270° spinal decompression is usually performed with large-diameter 18- and 20-mm titanium mesh. Given the difficulty in implantation of large titanium mesh during the operation, the long operation time, and the excision of more vertebral cancellous bone in the use of large titanium mesh, the authors’ hospital adopted limited spinal canal decompression with a 13-mm small diameter titanium mesh implanted, and the vertebral body was fixed in long segments. The advantages of the surgical method are that the titanium mesh with a small diameter of 13 mm is easier to implant, requires less resection of the injured vertebra, leads to a faster postoperative recovery of the patients, and significantly reduces operation time and blood loss. Second, although the titanium mesh with a diameter of 13 mm was small, the posterior column of the injured vertebra was fixed with a long fixation rod, which reduced the compression stress of the anterior column. Besides, imaging results showed no incision settlement of the vertebral body caused by the titanium mesh. Third, when a titanium mesh with a smaller diameter is used, the upper nerve roots are loose, thereby avoiding excessive traction of the nerve roots by the implanted titanium mesh. Some domestic scholars pointed out that the posterior internal fixation methods for thoracolumbar burst fractures include long and short vertebral segment fixation. The short vertebral segment internal fixation is more effective in reducing stress concentration in adjacent segments, while the long one is superior in fracture reduction, restoration of kyphosis, and reconstruction stability (18). According to the imaging results of the patient's follow-up, the anterior edge height of the injured vertebra was not lost, the vertebral morphology was complete, and the physiological curvature was good. There was no fracture of screws or titanium rods, and the fusion of the titanium mesh bone graft was good. After successful bone graft fusion for thoracolumbar burst fractures, removing pedicle screw rods is of great help to improve the patients’ low back pain and dysfunction, which might be related to the recovery of inter-segmental mobility (19). In the present study, not all patients had their screws removed. Generally, the screws of the anterior fusion segment cannot be removed but can be removed in some patients for psychological reasons. Depending on the level of injury to the posterior column of the spine, especially the extent and extent of injury to the ligamentous complex, the decision to remove the screws across the anterior fixation segment can be made. For some patients with posterior ligament injury, interlaminar bone grafting was performed across the fusion segment in consideration of long-term stability. The scope of screw fixation across the fusion level and whether to remove the screw has not been reported in domestic and foreign literature, and we were relatively cautious in removing the screw. This issue should be addressed in future studies.

This study has limitations. The sample size was small and from a single center, severely limiting the generalizability of the results. Bias due to local perioperative management cannot be excluded. Even though consecutive patients were selected, the inclusion/exclusion criteria might lead to a selection bias. As a retrospective study, the analyzable data were limited to those in the patient charts. No control group could be included.

In conclusion, spinal canal decompression and 13-mm titanium mesh implantation in the treatment of TLBF can achieve one-stage spinal canal decompression and three-column reconstruction. The curative effect is satisfying. This smaller mesh could be appropriate for younger patients with healthy bone structures. Additional studies are necessary to determine the patients who might benefit the most from using the 13-mm mesh.

## Data Availability

The original contributions presented in the study are included in the article/**Supplementary Material**, further inquiries can be directed to the corresponding author.
