# Supplementary material for: Clinical effect of limited posterior decompression and 13-mm titanium mesh implantation on severe thoracolumbar burst fractures: A case series
Source: Front Surg. 2023 Mar 16;10:1132569. doi: 10.3389/fsurg.2023.1132569 (PMC10060508; doi:10.3389/fsurg.2023.1132569)
Supplement: Supplementary file 1 [file Datasheet1.docx]

**Supplementary materials**

**Surgical methods**

After successful anesthesia, the abdomen was placed in the prone position with a U-shaped pad and made sure it was suspended. After fluoroscopy positioning and centering on the injured vertebral body, a posterior midline incision was made to expose the two upper and lower vertebrae of the injured vertebra. The five pedicles, including the injured vertebra, were exposed after a layer-by-layer incision. Eight screws were placed into the two upper and two lower vertebrae to the injured vertebra (Supplementary Figure S1). The lamina of the injured vertebra and the inferior articular process on one side of the injured vertebra were resected for decompression and buffer space. Part of the lamina on one side of the upper vertebra and the inferior articular process of the upper vertebra of the injured vertebra were resected to expose the dural sac (Supplementary Figure S2A). The dural sac was protected during the operation. The cancellous bone in front of the spinal canal was removed with a narrow bone knife and a small sharp scraper (Supplementary Figure S2B). A sneaking tunnel was excavated. A boot bone chisel was used to press the bone in front of the dural into the empty tunnel in front, thus ensuring the safety of the spinal cord. The vertebral body was cut at the inner edge of the pedicle of the injured vertebra with an osteotome, and then the pedicle was pressed to the outside to expose the vertebral body behind the endorachis. An osteotome and spatula were used to remove the part of the vertebral body that protruded into the spinal canal, the decompression areas were expanded, and an L-shaped bone hammer was used to hammer the contralateral spinal canal bone forward. Then, a probe and a nerve dissector were used to ensure that the spinal canal decompression was adequate and the decompression was completed (Supplementary Figure S2C).

The two upper and lower pedicles of the side contralateral to the decompression side were fixed and extended to expose and protect the upper and lower nerve roots on the decompression side. The endplate spatula was used to scrape off part of the endplate cartilage, and the titanium mesh was placed after measurement (Supplementary Figure S2D). Fluoroscopy was used to confirm that the posterior edge of the vertebral body had no obvious bone block protruding backward, the titanium mesh was in a good position, and bilateral pedicle screws were compressed. The second fluoroscopy was used to confirm that the titanium mesh was well attached to the endplate. The 0.9% NaCl-H_2_O solution preheated at 37°C was used to rinse the incisions. A negative pressure drainage tube was placed in the incision. Finally, the incision was sutured layer by layer.


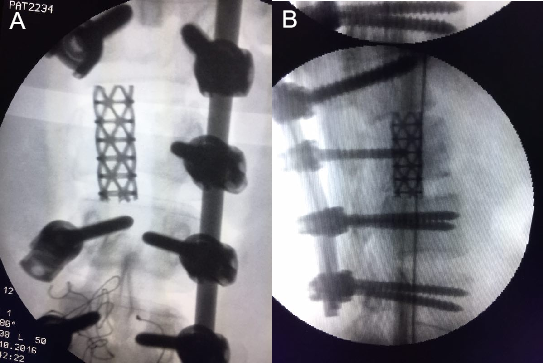


**Supplementary Figure S1.** Intraoperative fluoroscopy imaging. (A) Anterior perspective view of the titanium mesh and internal fixation device during surgery (digital X-ray). (B) Lateral view of the titanium mesh and internal fixation device during surgery (digital X-ray).


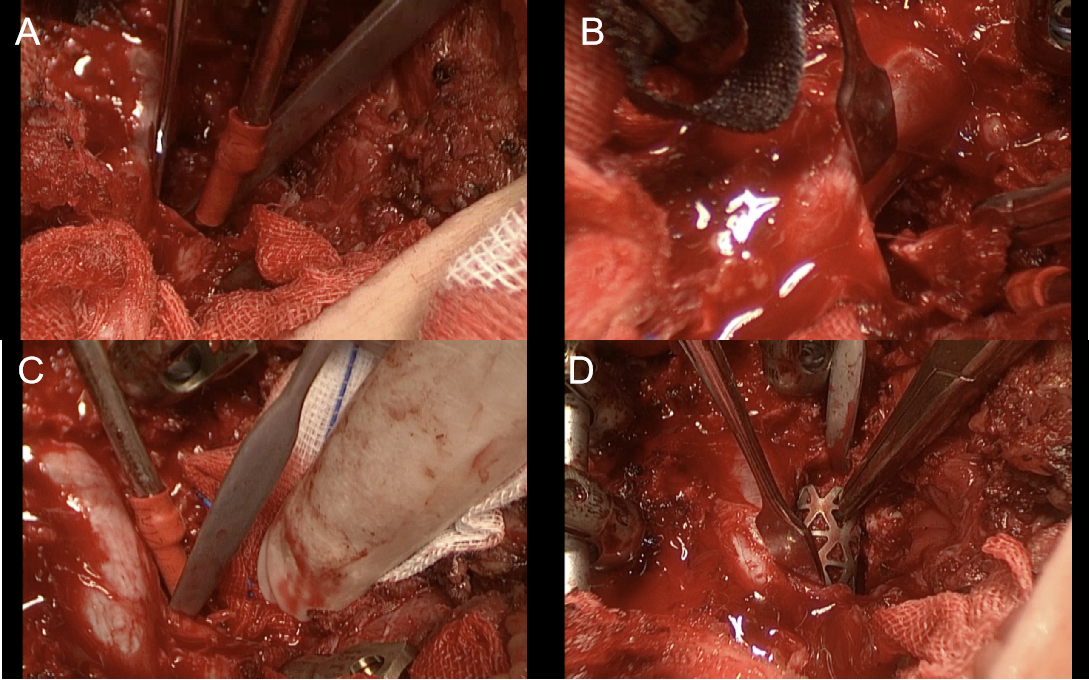


**Supplementary Figure S2.** Images of the key procedures during the operation. (A) Bone cutting the cone bone block. (B) Bone block of the injury cone. (C) Full exposure of the nerve roots (D) The 13-mm titanium mesh is placed.
